# Implicit IAT Measures and Neurophysiological fNIRS Markers in Response to High-Engagement Advertising

**DOI:** 10.3390/s23094332

**Published:** 2023-04-27

**Authors:** Michela Balconi, Martina Sansone, Carlotta Acconito

**Affiliations:** 1 International Research Center for Cognitive Applied Neuroscience (IrcCAN), Catholic University of the Sacred Heart, 20123 Milan, Italy; 2 Research Unit in Affective and Social Neuroscience, Department of Psychology, Catholic University of the Sacred Heart, 20123 Milan, Italy

**Keywords:** consumer decision making, implicit measures, neural correlates of attitudes, fNIRS, IAT

## Abstract

Self-report measures partially explain consumers’ purchasing choices, which are inextricably linked to cognitive, affective processes and implicit drives. These aspects, which occur outside of awareness and tacitly affect the way consumers make decisions, could be explored by exploiting neuroscientific technology. The study investigates implicit behavioural and neurovascular responses to emotionally arousing and high-engagement advertisements (COVID-19 content). High-engagement advertisements and control stimuli were shown in two experimental sessions that were counterbalanced across participants. During each session, hemodynamic variations were recorded with functional Near-Infrared Spectroscopy (fNIRS) of the prefrontal cortex (PFC), a neurophysiological marker for emotional processing. The implicit association task (IAT) was administered to investigate the implicit attitude. An increase in the concentration of oxygenated haemoglobin (O2Hb) was found for the high-engagement advertising when this category of stimuli was seen first. Specular results were found for deoxygenated haemoglobin (HHb) data. The IAT reported higher values for highly engaging stimuli. Increased activity within the PFC suggests that highly engaging content may be effective in generating emotional arousal and increasing attention when presented before other stimuli, which is consistent with the higher IAT scores, indicating more favourable implicit attitudes. This evidence suggests that the effectiveness of highly engaging advertising-related messages may be constrained by the order of advertisement administration.

## 1. Introduction

Every year, about 76% of newly launched products are taken off the market within 12 months from their launch, as stated in a report conducted by Nielsen in 2015 [1]. Such daunting results do not spare products that were the target of prior market research. However, it is perhaps not surprising to find such a gap between marketing forecasting and actual product success, as traditional marketing techniques widely rely on surveys and interviews.

Research has provided abundant evidence that self-report measures can be misleading at times due to the discrepancy between explicit and implicit mental processes [2,3,4]. Questionnaires, surveys, focus groups, and interviews are well-suited instruments for investigating consumers’ overt opinions, which can be easily expressed out loud, yet this feature shrinks the field of the investigation to cognitive aspects that are consciously processed. However, purchase choices are inextricably linked to affective processes and implicit drives that should not be dismissed if the aim is to achieve a comprehensive understanding of the consumer’s decision-making process [5].

Indeed, there are several reasons for which explicit responses may fail to reflect the consumers’ deepest thoughts and resultant behaviours. Social desirability and social pressure are major issues, since consumers may be unwilling to disclose their genuine opinion about a product (or advertising) when intimate or prejudice-prone themes are investigated [6,7], or they may be ashamed of expressing their preference for products or brands because of how the preference reflects onto their self-image [8]. In addition, when compiling surveys after testing a commercial or a product, customers may simply not be able to faithfully retrace in hindsight how they felt at different time-points of the experience, introducing memory biases (such as primacy or recency effects).

Generally, numerous emotional, evaluative, and spontaneous processes occur outside of awareness and tacitly affect the way consumers make decisions without them even noticing [2,9,10]. Thus, in this sense, it is possible to assume that when people are engaged in watching an advertisement, emotional stimuli might pass through the filter of consciousness. At this point, a more direct analysis of implicit measures is noteworthy.

The latest evidence, however, challenges key elements of the theories of emotional motivation, particularly the notion that emotional cues automatically cause behavioural responses in the observer [11,12]. According to this new line of thought, specifically, emotional facial expressions [13,14,15,16] and body postures [17] elicit a consistent and replicable behavioural effect only when they are relevant to the participants’ goals. This is, therefore, a context-dependent effect of emotions in which the response to emotional stimuli is linked to conscious appraisal [18].

To date, novel approaches have been devised to offer more objective measures of implicit processes and to overcome the limits of direct, introspective verbal assessments. On one hand, cognitive-neuroscience-inspired methods (as Spence defines them) [19] have been successfully employed to this end. Above all, the implicit association task (IAT) has been deemed a reliable, powerful tool for assessing implicit associations between two target categories (in this research field, usually a brand or product and the pleasant/unpleasant evaluation) [20,21] under the assumption that the stronger the mental association, the faster the behavioural response when the two categories share the same response key [22]. A strong association between the target object and the positive evaluation pole represents a positive implicit attitude towards the object. Numerous studies have shown that explicit (questionnaire-based) and implicit (IAT-based) measures of the same association are frequently poorly correlated or even incongruent [23], highlighting the fact that a consumer may simultaneously hold two distinct evaluations of an object: one that is consciously reasoned and one that remains excluded from the consumer’s awareness. Interestingly, in these cases, implicit measures are generally better predictors of spontaneous behaviour than explicit responses. Specifically, when the two measures are incongruent, people under time pressure tend to select the implicitly preferred object (e.g., a brand) rather than the brand they explicitly stated to prefer [2]. When the two measures are poorly correlated, implicit responses increase the accuracy of the predictive model of consumer behaviour compared to explicit measures alone, providing a significant contribution to the model independently from the explicit measures [24].

On the other hand, the advent of consumer neuroscience has opened up the possibility of investigating the neural correlates of consumer decision making, providing consumer studies with methods, paradigms, and theoretical insights derived from neuroscience. In recent years, numerous studies have adopted neuroimaging techniques to disentangle the neural underpinning of affective and cognitive processes involved in the consumer experience that were impenetrable to classical methods [25]. It is interesting that several studies have demonstrated that neural responses can be valid predictors of consumer preferences and behaviour when compared to self-reports. For instance, an influential experiment by Knutson and colleagues [26] showed that activity within the nucleus accumbens (NAcc), the right insula, and the medial prefrontal cortex (mPFC), recorded via functional magnetic resonance imaging (fMRI), could predict purchase choices more accurately than subjective ratings alone in relation to product preference and willingness to pay. Indeed, the NAcc and the insula are implied in anticipating gains and losses, respectively, while the mPFC is likely responsible for integrating gains and losses and taking stock of the trade-off. However, this behavioural process of evaluating resources and choices turns out to also be linked to the dopaminergic system. Indeed, it has been shown that dopaminergic signals implement this mechanism by providing information to the PFC about the need to appropriately update goal representations [27,28].

Additionally, a number of studies have also highlighted that neural responses to marketing stimuli, collected from small experimental samples, can be generalized to the population and predict commercial success. For instance, increased activity in the NAcc, measured from a small sample of teenagers listening to newly released songs, could predict the cultural popularity in terms of commercial sales of the songs within the following three years, whereas subjective likeability evaluations could not [29]. Similarly, Boksen and Smidts [30] noted that not only did beta oscillations within the mPFC predict individual preferences for movie trailers beyond explicitly stated preferences but gamma oscillations in the frontal areas were also related to the commercial success of the movies, as measured by box office results. Moreover, a remarkable fMRI study by Kühn and colleagues [31] adopted a composite neural measure that combined information from the most relevant areas involved in purchase decision making (i.e., the NAcc, medial orbitofrontal cortex, amygdala, hippocampus, inferior frontal gyrus (IFG), dorsomedial prefrontal cortex (dmPFC), dorsolateral prefrontal cortex (DLPFC), and the insula) to assess the effectiveness of six different advertisements. Notably, the composite neural measure demonstrated the highest predictive validity as it forecasted the actual in-store product sale according to the different advertisements with the highest accuracy. Conversely, explicit measures yielded the lowest predictive power. 

Regarding the involvement and activation of the PFC, it is interesting to note that it also plays an important role in emotional contexts related to social touch [32,33]. Moreover, social touch is connected not only with prefrontal activation but also with parietal patterns, suggesting that the interaction between prefrontal systems regulating emotions and parietal, mirror-like systems may be the source of the positive effects of social touch [34].

Although there is encouraging evidence for the future application of neurometrics to the study of the consumer mind, a deeper understanding of the role of implicit processes must be pursued nonetheless. Indeed, despite the undeniably relevant role of implicit components, decision making generally takes the form of a complex reiterative process that combines stages of both explicit and implicit reasoning [35,36]. Therefore, according to this perspective, the effect of a given behaviour, which is the result of emotional and cognitive processes that occur both while the object/task is the focus of conscious attention and when attention is directed elsewhere, affect the mode and purpose of the subsequent decision in turn. Therefore, it is a circular pathway.

In light of this evidence, the present study sought to understand the role of some implicit measures (i.e., the IAT and Self-Assessment Manikin (SAM)) and relevant neurophysiological markers. Specifically, the study aimed to investigate the effect of different emotional brand commercials on implicit attitudes towards the brand, assessed using the IAT, their levels of valence and arousal, assessed using the SAM, and the neural correlates of the advertising content elaboration, recorded via functional near-infrared spectroscopy (fNIRS). One of the latest entries in consumer neuroscience studies [37,38], fNIRS is now acknowledged as a valid, non-invasive, and portable instrument that is poorly sensitive to motor artifacts and is suitable for measuring the functional activity of cortical brain regions based on an assessment of oxygenated and deoxygenated cerebral haemoglobin concentrations [39]. 

Neural activity from the PFC was recorded in light of the abundant evidence suggesting its implication in attitude formation [40], consumer preference, and decision and emotional processing [41,42]. However, it is worth noting that the emotional regulation network does not consist of the PFC alone but also involves a cortical and a subcortical system [43]. This network includes, for example, the amygdala, which is responsible for monitoring the emotional value of stimuli and sending feedback to sensory pathways [42]; the ventral striatum, a region implicated in encoding the reward values of stimuli, learning, and predicting positive outcomes [44]; the anterior cingulate cortex, which is particularly relevant to the cognitive control of emotions [45]; and the insula, which allows for the representation of body states associated with emotions [43].

Indeed, in the current study, emotional commercials that could display content related or unrelated to the COVID-19 pandemic were selected under the assumption that a worldwide dramatic experience may evoke a more vivid and intense affect and higher engagement [46,47] compared to commercials unrelated to the pandemic, and that this could have a differential effect on the effectiveness of brand communications on implicit dimensions.

We hypothesized that COVID-19 advertisements could elicit greater emotional engagement when compared to ads that were unrelated to COVID-19. For this reason, we expected to find increased neural activity within the PFC during the COVID-19 commercials compared to control advertisements as a neurophysiological marker for emotional processing and motivational dispositions, which are known to recruit the PFC [40,41]. In addition, this emotional engagement and impact were expected to be supported by the self-report SAM measure, so it would be possible to assume greater arousal for COVID-19-related stimuli. Furthermore, as participants in the study saw both COVID-19-related and unrelated advertisements one after another, in a counterbalanced order, the study also intended to test the order effect, namely, whether the exposure to the COVID-19 condition, which appeared first in the order, could have a successive effect on brand evaluation, which could possibly be echoed in the following non-COVID-19 condition. Indeed, under specific circumstances, strong emotional content can have a longer-lasting effect on memory compared to mild emotional stimuli [48]. 

Moreover, we expected that the COVID-19 ads might lead to more favourable implicit attitudes toward the brand, as measured by the IAT. Indeed, prior studies have shown that under certain conditions, emotionally negative stimuli may be beneficial to the effectiveness of advertisements [49,50], perhaps by gaining consumers’ attention to a greater extent and acting as an emotional lever that successfully tugs the consumers’ heartstrings [40,41]. Although other research studies have already assessed the neurophysiological response to COVID-19 stimuli [46], the present study aimed to investigate the effect of COVID-19 stimuli in order to gain further insights from implicit behavioural data. Therefore, a more direct comparison with new implicit measures was included in the present work. 

Finally, we expected the neural markers of motivational dispositions to correlate with the IAT indices. In fact, evidence regarding the neural correlates of implicit attitudes is beginning to emerge in a variety of research areas [40,51,52,53], some of which have revealed the involvement of the prefrontal cortex [40,51,53]. Hence, we expect positive (vs. negative) implicit IAT evaluations (i.e., more positive scores indicate a more favourable attitude toward the brand) to correlate with left (vs. right) PFC predominance [54].

## 2. Materials and Methods

### 2.1. Participants

Twenty Italian participants, aged 25.47 years old on average (a range of 20–30 years old; Mage = 24.98; SDage = 2.02; 14 females), took part in the experiment. Any participant reporting a neurological or psychopathological disorder, head injury, epilepsy, or who was under psychopharmacological treatment was excluded from the study. Participants were also screened for post-traumatic stress symptomatology related to the COVID-19 pandemic via the COVID-19-PTSD questionnaire [55] to exclude the possibility of participants scoring over the clinical cut-off. All participants included were right-handed and had normal-to-corrected eyesight. The participants were mostly college students recruited within the Catholic University of the Sacred Heart, Milan, Italy. They all provided written informed consent prior to participating in the study, and no economic compensation was provided. The research protocol followed the principles of the Declaration of Helsinki (1964) and was approved by the Ethics Committee (Approval code: 2021 TD—for thesis dissertation; approval date: 21–22) of the Catholic University of the Sacred Heart, Milan, Italy.

### 2.2. Stimuli

Six video commercials were selected as stimuli for the present study. All commercials were produced by Nike, a popular sportswear brand with an international reputation. Nike’s communications are notoriously famed for their inspirational tone of voice, which cleverly combines emotional and motivational elements in the advertising to promote empowering messages while selling the brand image at the same time. For the purpose of the study, three of the selected commercials (namely “Play for the World”, “You Can’t Stop LA”, and “You Can’t Stop Us”—see Supplementary File for links to advertisements) contained explicit visual references to COVID-19, playing with the analogy between athletes’ endeavours to overcome barriers to their victory and the societal fight against the pandemic. Moreover, three additional commercials were selected (“What’s your motivation?”, “You can’t be stopped”, and “Steps”—see Supplementary File for links to advertisements) as control stimuli which displayed the typical elements of Nike’s communication without any reference to the COVID-19 pandemic. The content of these commercials broadly urges the audience to believe in the deepest motives to orient their efforts towards success, reminding the listeners that commitment and dedication are values that can truly guide humanity through difficulties and towards achievements. At the same time, however, these videos were also made for commercial purposes, as they aimed to increase the public’s willingness to buy sportswear during the pandemic, when sports had to be played at home. The stimuli, which were validated in a prior exploratory study [45], had a frame rate of 24 fps and lasted 60 s each. A detailed description of each stimulus can be found in Balconi and colleagues’ study [45].

### 2.3. Behavioural Measure: Implicit Association Task (IAT)

The IAT [22] is a reaction-time-based computerized task that requires participants to assign four categories of stimuli to the corresponding labels. Two categories represent the target concept that needs to be evaluated (i.e., Nike) and a contrasting target concept (i.e., other sportswear brands), while the other two categories represent two contrasting attributes (i.e., pleasant vs. unpleasant words). In our task, stimuli were devised so that the brand-related stimuli were pictures of sportswear items (e.g., a Nike hat vs. a Puma hat). Conversely, the evaluative stimuli were displayed as a set of positively or negatively qualified adjectives (e.g., “good” vs. “bad”). The stimuli appeared randomized (according to the standardized procedures suggested for the standardized version of the IAT [56]) in the centre of the screen, and participants were asked to respond as fast as possible to assign the stimulus to the correct label by pressing the assigned computer key. Specifically, the names of brands were shown first: two brands were shown on the screen (one was always Nike), one on the right and one on the left. Participants were required to press a left/right button according to their preference (e.g., the “E” key on the keyboard for left-side responses and the “I” key for right-side responses). Immediately after this choice, two adjectives (“good” or “bad”) were shown, one on the right and one on the left, and subjects had to press the corresponding button (e.g., the “E” key on the keyboard for the left-side responses and the “I” key for the right-side responses). The IAT is based on the assumption that when two concepts (one target and one evaluative concept) are mapped onto the same response key, participants respond faster if the two concepts are implicitly associated, whereas they respond slower if the two concepts are not. The strength of the mental association between the target concept and the attribute is estimated according to the response times (reaction times). D-scores were calculated according to the improved scoring algorithm [56] such that more positive scores indicated a more favourable attitude toward Nike. E-Prime 2.0 software was used to program the task, and it was run on a 15′’ computer notebook (Lenovo, MS Windows 7) with a QUERTY keyboard.

### 2.4. Self-Report Measure: Self-Assessment Manikin (SAM) Scale

The SAM scale [57,58], which is a nonverbal pictorial rating technique that quantifies the valence and arousal of an individual’s emotional response to a particular stimulus, was adopted for self-report data collection. Using a 5-point Likert scale represented by five different pictures, the subject was asked to indicate the degree of emotional impact (1: low impact; 5: high impact) and the level of negativity–positivity felt upon viewing the video (1: negative; 5: positive). 

### 2.5. Procedure and Experimental Design

**Figure 1:**
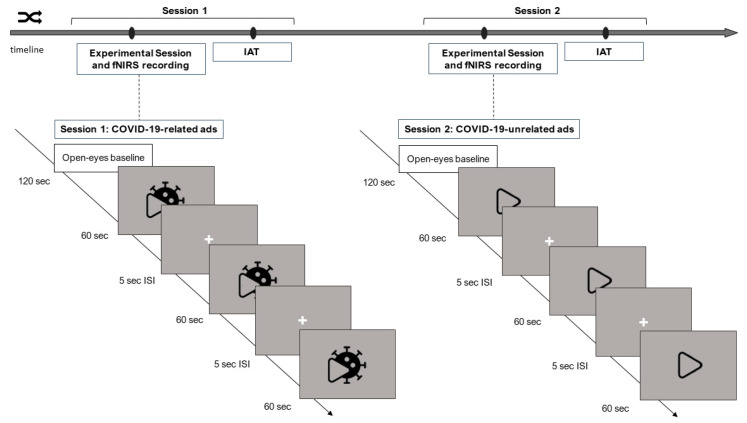
Experimental procedure. The figure exemplifies the timeline of the experiment when the “COVID-19 first” order was administered. During session 1, three COVID-19-related advertisements were displayed. The second session took place 15 days after, and three advertisements unrelated to COVID-19 were shown. At the beginning of each experimental session, a 120 s baseline of neural activity at rest was recorded via fNIRS. Hemodynamic changes were recorded during the stimuli administration. Stimuli were presented in a randomized order within each experimental session. The stimuli lasted 60 s and were alternated with a 5 s inter-stimulus interval (ISI). At the end of both experimental sessions, the IAT was administered.

A within-subjects experimental design was adopted in the present study. All participants took part in two experimental sessions, which were scheduled 15 days apart. In one of the two sessions, the participants were administered the three COVID-19-related commercials (COVID-19 condition), while in the other session, the three control stimuli were displayed (control condition). The order of the two conditions was counterbalanced across participants (COVID-19 first vs. COVID-19 after). During each session, participants sat comfortably in a moderately darkened room at about 80 cm from the monitor screen. The fNIRS setup was mounted on the participant’s head, and baseline hemodynamic activity was recorded for 2 min while participants were required to keep their eyes open at rest. The three videos were then presented on the screen in a randomized order while the hemodynamic activity was continuously recorded. Following each video, a 5s inter-stimulus interval was displayed, during which a fixation point at the centre of the computer screen was presented prior to the next video. At the end of each experimental session, the IAT was administered (Figure 1). Each experimental session lasted approximately 1 h. 

### 2.6. fNIRS Data Analysis

**Figure 2:**
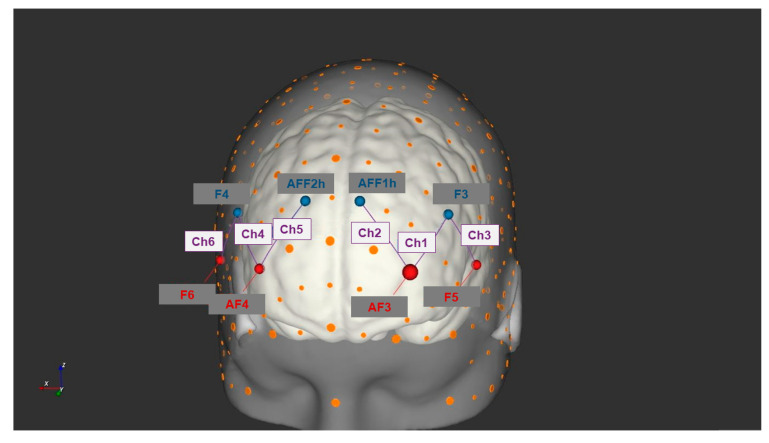
fNIRS setup. A matrix of 6 channels was displayed over the prefrontal areas to measure O2Hb and HHb variations. The 4 emitters were positioned at AF3–AF4 and F5-F6 (red), and the 4 detectors were placed at AFF1h-AFF2h and F3-F4 (blue). The 6 resulting channels (purple) were arranged as follows: Ch1 (AF3–F3), Ch2 (AF3–AFF1h), Ch3 (F5–F3), Ch4 (AF4–F4), Ch5 (AF4–FF2h), and Ch6 (F6–F4). The software nirSite (version 2.0) (NIRx Medical Technologies LLC) was used to create the 3D render.

The NIRScout System (NIRx Medical Technologies, LLC, Los Angeles, CA, USA) was used to record the variations in the concentration levels of oxygenated haemoglobin (O2Hb) and deoxygenated haemoglobin (HHb). A 6-channel array of optodes (4 emitters and 4 detectors) was used, encompassing the prefrontal region. An fNIRS Cup was used to arrange the optodes according to the 10/5 international system [59]. Specifically, emitters were placed at AF3–AF4 and F5–F6, whereas detectors were placed at AFF1–AFF2 and F3-F4. The distance between the paired emitters and detectors was kept at 3 cm, and two infrared wavelengths were used (760 and 850 nm). The channels were arranged as follows: Ch1 (AF3–F3), Ch2 (AF3–AFF1h), Ch3 (F5–F3), Ch4 (AF4–F4), Ch5 (AF4–AFF2h), and Ch6 (F6–F4) [60,61]. The anatomical correspondence of each channel was identified with the Automated Anatomical Labeling toolbox from fOLD software (version 2.2.1) (fNIRS Optodes’ Location Decider) [62]. The following correspondences between channels and Broadmann areas were identified: Ch1 and Ch4 overlap with the left and right DLPFC (BA 46); Ch2 and Ch5 overlap with the left and right frontopolar area (BA 10) and a portion of the left and right DLPFC (BA 46); Ch3 and Ch6 overlap with the left and right pars triangularis of Broca’s area (BA 45) (Figure 2).

NIRStar Acquisition Software (version 12.4) was used to continuously record changes in the concentrations of oxygenated (O2Hb) and deoxygenated (HHb) haemoglobin during both the baseline and the experimental phases. Signals from the 6 NIRS channels were acquired at a sampling rate of 6.25 Hz and were analysed and transformed using nirsLAB software (v2014.05; NIRx Medical Technologies LLC, 15 Cherry Lane, Glen Head, NY, USA) according to their wavelength and location. The procedure resulted in mmol∙mm values for changes in oxygenated and deoxygenated haemoglobin concentrations for each channel. Then, each channel’s raw O2Hb and HHb data were digitally bandpass filtered at 0.01–0.3 Hz. The mean concentration of each channel was then computed by averaging the data across trials for the two conditions. The effect size of each condition was calculated based on the mean concentrations in the time series for each channel and subject. The effect sizes (Cohen’s d) were computed as the difference between the concentration means at the baseline (m1), collected for 120 s with eyes open at rest, and at trial (m2), which was identified as the single period of viewing a single stimulus, divided by the baseline standard deviation(s). The following formula was used: d = (m1–m2)/s. The effect sizes computed from the six channels were then averaged to maximize the signal-to-noise ratio. Indeed, although the raw fNIRS data were initially relative values that could not be directly averaged across participants or channels, the normalized effect size data could be averaged regardless of the unit because effect size is unaffected by differential pathlength factors (DPF) [63].

### 2.7. Statistical Data Analysis

Three sets of analyses were performed with respect to neurophysiological (O2Hb and HHb mean values), implicit behavioural (IAT), and self-report (SAM) measures.

For neurophysiological measures, two ANOVAs were performed. First, a two-way ANOVA with the order (2: COVID-19 first vs. COVID-19 after) as the between-subject factor and the condition (2: COVID-19 vs. non-COVID-19) as the independent within-subject factor was applied. Then, a three-way ANOVA with the order (2: COVID-19 first vs. COVID-19 after) as the between-subject factor and the condition (2: COVID-19 vs. non-COVID-19) and Channel (6: Ch1, Ch2, Ch3, Ch4, Ch5, Ch6) as the independent within-subject factors was performed.

For IAT d’ values, a mixed repeated measures ANOVA was performed using the condition (2: COVID-19 vs. non-COVID-19) as the independent within factor and the order of the two conditions as an independent between factor (2: COVID-19 first vs. COVID-19 after). Pairwise comparisons were used to check simple effects for significant interactions. For all the analyses performed on O2Hb, HHb, and IAT, pairwise comparisons were adopted to assess simple effects for significant interactions, and the Bonferroni correction was used to reduce biases in the repeated comparisons. For all ANOVAs, the degrees of freedom were corrected where appropriate via the Greenhouse–Geisser epsilon. The kurtosis and asymmetry indices were considered to determine the normality of the data distribution, and the size of statistically significant effects was estimated by using partial eta squared (η^2^) indices.

For SAM, arousal and valence subjective ratings were analysed with two separated repeated measure ANOVAs, taking the order (2: COVID-19 first vs. COVID-19 after) as the between-subject factor and the condition (2: COVID-19 vs. non-COVID-19) as the independent within-subject factor.

## 3. Results

### 3.1. fNIRS

The ANOVA performed on the oxygenated haemogloblin (O2Hb) D scores yielded the following results. 

**Figure 3:**
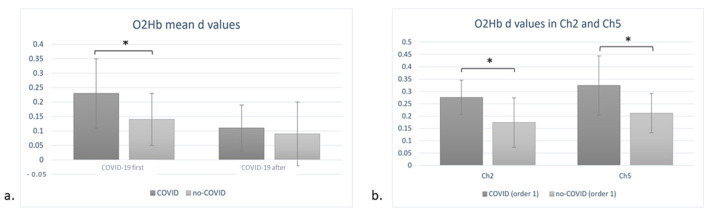
(**a**,**b**). Oxygenated haemogloblin (O2Hb) variations. (**a**) The interaction effect order × condition was significant (F(1, 18) = 7.65, *p* = 0.01) for O2Hb concentrations, highlighting increased O2Hb levels under the COVID-19 condition compared to the non-COVID-19 condition only when the order ”COVID-19 first” was administered. (**b**) A three-way interaction of order × condition × channel (F(4, 27) = 5.67, *p* = 0.01) highlights that when the ”COVID-19 first” order was administered, Ch2 and Ch5 displayed significantly higher O2Hb mean concentrations for the COVID-19 condition than the non-COVID-19 condition. All data are represented as mean ± SE; all * statistically significant differences, with *p* ≤ 0.05.

A significant interaction effect of order × condition was found (F(1, 18) = 7.65, *p* = 0.01, η^2^ = 0.459). Indeed, under the COVID-19 condition, participants that followed the “COVID-19 first” order showed overall significantly higher levels of O2Hb concentration when compared to the non-COVID-19 condition. Conversely, no significant difference was found for participants that were administered the “COVID-19 after” order (Figure 3a). Moreover, a significant three-way interaction, order × condition × channel, was revealed (F(4, 27) = 5.67, *p* = 0.01, η^2^ = 0.432). Specifically, for participants that followed the “COVID-19 first” order, Ch2 (AF3–AFF1h) and Ch5 (AF4–AFF2h) displayed significantly higher mean concentration values under the COVID-19 condition with respect to the non-COVID-19 condition. No significant interactions were found for participants that followed the “COVID-19 after” order (Figure 3b).

**Figure 4:**
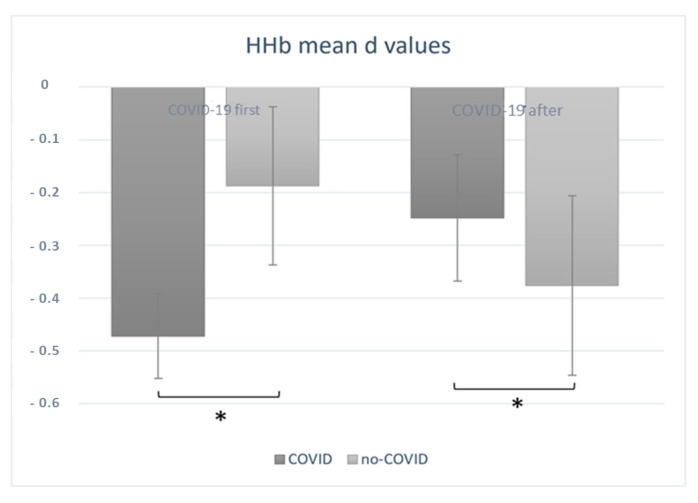
Deoxygenated haemogloblin (HHb) variations. The interaction effect of order × vondition was significant (F(1, 18) = 8.09, *p* = 0.01) for the mean concentration of HHb, suggesting that, when the “COVID-19 first” order was administered, the HHb concentration levels decreased under the COVID-19 condition when compared to the non-COVID-19 condition. Conversely, when the “COVID-19 after” order was administered, the HHb mean values significantly increased under the COVID-19 condition when compared to non-COVID-19 condition. All data are represented as mean ± SE; all * statistically significant differences, with *p* ≤ 0.05.

The ANOVA performed on HHb D scores yielded the following results. A significant interaction effect of order × condition was found (F(1, 18) = 8.09, *p* = 0.01, η^2^ = 0.589). Indeed, under the COVID-19 condition, participants that followed the “COVID-19 first” order showed overall significantly lower concentration levels of HHb when compared to the non-COVID-19 condition. In addition, for the participants that were administered the “COVID-19 after” order, the HHb mean values significantly increased under the COVID-19 condition when compared to the non-COVID-19 condition (Figure 4).

### 3.2. IAT

**Figure 5:**
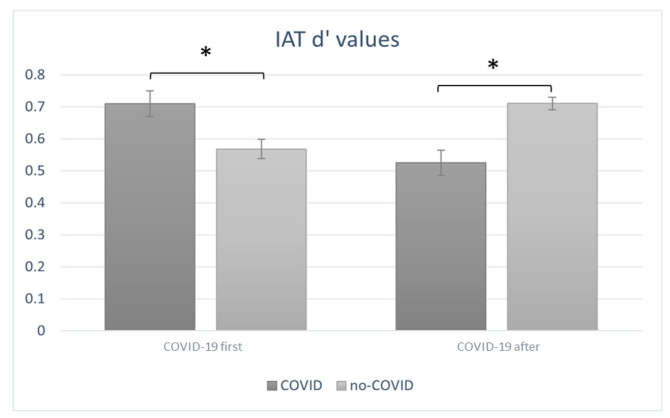
IAT results. The graph shows that a significant interaction effect of order × condition (F(1, 18) = 8.98, *p* = 0.01) was found for IAT behavioural results. Indeed, when the “COVID-19 first” order was administered, higher IAT scores were found following the COVID-19 condition as compared to the non-COVID-19 condition. Conversely, when the “COVID-19 after” order was administered, participants scored higher following the non-COVID-19 condition than following the COVID-19 condition. All data are represented as mean ± SE; all * statistically significant differences, with *p* ≤ 0.05.

The ANOVA carried out on the IAT d’ values highlighted a significant interaction effect of order × condition (F(1, 18) = 8.98, *p* = 0.01, η^2^ = 0.598). Indeed, when the “COVID-19 first” order was administered, participants scored higher d’ values when following the COVID-19 condition than following the non-COVID-19 condition. Conversely, when the the “COVID-19 after” order order was administered, participants scored higher d’ values following the non-COVID-19 condition than following the COVID-19 condition (Figure 5). Higher and positive d’ scores indicate a more favourable attitude toward the brand Nike.

As a result of the specific aim and objectives of this study, the results reported refer only to the participants’ preferences toward the Nike brand.

### 3.3. SAM

The ANOVA carried out for arousal ratings reported a significant main effect in the within-subject factor condition (F(1, 18) = 11.469, *p* = 0.003, η^2^ = 0.389). Post-hoc pairwise comparisons revealed higher arousal ratings for the COVID-19 condition compared to the non-COVID-19 condition (*p* = 0.003). No other main or interaction effect was significant. The ANOVA carried out for the valence ratings did not highlight significant main or interaction effects.

## 4. Discussion

The present study sought to investigate the implicit responses to COVID-19 emotional advertising. The results suggest that COVID-19 ads can be effective, but their effect is also constrained to the order effect. At the same time, this research, specifically the results of the SAM self-report data, suggest that arousal and not the valence of advertisements appear to impact behavioural and neural outcomes.

Indeed, when first considering the results from the “COVID-19 first” order, participants showed an increase in concentrations of oxygenate haemoglobin (O2Hb) under the COVID-19 condition—which was the first to be displayed—with respect to the non-COVID-19 condition. Specular results from the deoxygenate haemoglobin data (HHb) provide further support to this effect. Indeed, participants that were administered the “COVID-19 first” order showed a significant decrease in HHb values under the COVID-19 condition when compared to the following non-COVID-19 condition. Since a decrease in deoxygenate haemoglobin indicates heightened neural activity, the two neural markers combined together suggest a significant increase in prefrontal neural activity when the COVID-19 contents are displayed first, followed by a reduction in neural activity when the COVID-19-unrelated themes are presented thereafter. Such results are consistent with previous studies, suggesting a role of the PFC in emotional processing [64,65,66], which was expected to be recruited to a higher extent during the exposure to highly arousing COVID-19 stimuli compared to neutral stimuli. 

At the same time, it might be useful to highlight the role of the PFC in emotional resonance and embodiment, which are identifiable as two key aspects of emotional arousal. From the different definitions of emotional resonance, it can be defined as the ability to (i) know what another person is feeling [67], (ii) have the intention to respond compassionately to another person’s distress, and (iii) mimic what another person is feeling by responding with similar emotional behaviour [68]. Studies have demonstrated that prefrontal activations related to emotional resonance are amplified when people see the same emotionally significant events occur to psychologically similar models (humans, animals, and robots) [69,70,71]. Ionta and colleagues, for instance, pointed out how seeing pain in humans (who are psychologically closer to the participants) prompts higher activity in the precentral gyrus than viewing pain in animals and robots (who are psychologically farther from the participants) [72]. Similarly, when people see emotionally salient events happening to people closer to them, such as friends, the emotional resonance activity in the prefrontal cortex is stronger with respect to observing emotional circumstances involving distant people, such as strangers [73].

Regarding embodiment, which is defined as the feeling of being inside one’s physical body, it is important to emphasize that it represents a source of information in judgment and choice processes [74] and requires specific brain mechanisms [75]. Specifically, several studies have shown how the manipulation of facial expressions and postures can influence emotional reactions to stimuli, affecting physiological and cognitive responses, as represented by the activation of the prefrontal cortex [76,77,78]. For instance, it has been demonstrated that participants perceive a virtual bodies as being a part of their bodies after receiving synchronous visual–tactile stimulation (embodiment), and the prefrontal cortex (also recognized as the premotor cortex) is activated during emotionally significant vicarious somatosensation [79].From the findings regarding emotional resonance and embodiment, it could be assumed that the activation of the PFC found in this study may reflect an increase in the participants’ perceived psychological closeness to COVID-19-related content and somatosensory vicariousness for the human bodies shown in the videos with increased emotional arousal. This perspective may then offer further evidence as to why PFC activation was significantly greater for the “COVID-19 first” condition.

Moreover, it should be noted that for the “COVID-19 first” order, O2Hb concentration levels increased under the COVID-19 condition, specifically at Ch2 and Ch5, which were identified with the left and right frontopolar regions (BA 10) and a portion of DLPFC (BA 46). In this regard, the activity found at BA 46 is consistent with our hypothesis that COVID-19-related stimuli may have triggered a more extensive emotional engagement. Indeed, a wide evidence base supports the relevant role of DLPFC in emotional regulation—with specific reference to the regulation of negative affect [64]—and its mediating role in orienting attention towards emotionally salient stimuli, which eventually results in an increased efficiency of salient emotional stimuli processing [42].

Interestingly, as far as the activation found in BA 10 is concerned, this rostral portion of the prefrontal cortex was recently proposed as a crucial area serving as a “gateway” which deploys cognitive and attentional resources between internal mental representations elicited in response to environmental stimuli, mostly sensory-related stimuli, and mental representations that occur independently from the environment [80]. A possible explanation for the reported activity at BA 10 is that the pandemic stimuli may have triggered an additional metacognitive effort, perhaps generating a conflict regarding the need to orient the resources toward the advertising stimuli or toward the internal independent representations (i.e., thoughts) that are likely to be prompted in the first place by the reference to a world-wide dramatic experience that has been shared by virtually the whole of humankind. 

On the other hand, when the “COVID-19 after” order was administered, the O2Hb results did not differ under the two experimental conditions. However, the HHb mean concentrations did. Indeed, higher HHb values were found under the COVID-19 condition when it was presented following the non-COVID-19 condition, highlighting an overall diminished neural activity within the PFC. Taking together the findings from the haemodynamic variations in the “COVID-19 first” order and two, it is possible to hypothesize that a sort of carry-over effect may have occurred, which could explain the differential response to COVID-19 stimuli when the stimuli were administered in different orders. Indeed, when COVID-19 stimuli were presented following the stimuli unrelated to COVID-19, the prefrontal activity did not appear to engage in the attentional and emotional processing of COVID-related information as extensively as what occurred when the pandemic stimuli were presented in the first place. A possible explanation for such an effect is that the previous presentation of non-COVID-related stimuli may have flattened the perception of the emotional components of pandemic-themed advertisings. The extant literature has provided abundant evidence that repeated exposure to advertising may undermine the effectiveness of the advertisement [81,82,83], outlining an inverted U-shaped curve of the effect of repetition on advertisement effectiveness [84]. Although the debate regarding the factors responsible for this effect is still ongoing, there is some solid evidence that the repetition effect is closely related to the level of arousal evoked by the advertisement, suggesting that multiple repetitions may lower the perceived arousal. Lower arousal and emotional activation could then reflect a diminished capacity to elicit liking responses and favourable attitudes towards the ad [85]. Thus, in the present study, although the COVID-19 ads resulted in effective emotional engagement when presented in the first session, they may have lost their efficacy when they were preceded by the repetition of other advertising stimuli of the same brand. In fact, excessive repetition may result in redundancy or boredom, which are deemed to negatively affect advertisements’ effectiveness [86].

Notably, in the “COVID-19 after” order, the effect was not specifically localized at BA 10 and 46, which might perhaps suggest that although the COVID-19 unrelated advertisements may have triggered emotional processing to a greater extent when compared to the following presented COVID-19 commercials, they did not recruit particularly pronounced metacognitive conflicts for attentional resources [87]. The activation during non-COVID-19 commercials was indeed extended to the overall PFC. However, the reasons for such a differential localization of the neural activation pattern should be further explored.

Finally, the implicit behavioural results gained through the IAT were revealed to be consistent with the neurophysiological data. Indeed, considering the “COVID-19 first” order, participants showed more favourable attitudes toward the brand following the administration of COVID-19 stimuli shown first when compared to the non-COVID-19 stimuli displayed thereafter. Conversely, in the “COVID-19 after” order, participants showed more favourable attitudes toward the brand following the vision of non-COVID-1-related commercials when they were first exhibited compared to COVID-19 stimuli presented thereafter. Hence, the carry-over effect may also extend to the behavioural findings. Although further research is needed to reach a better understanding of the factors that may have prompted such an effect, the present results suggest the activity of the prefrontal cortex may represent a valid neural marker for the emotional and attentional processes that are likely to support the development of favourable implicit attitudes. 

However, as pointed out in the Section 1, it is necessary to keep in mind that recent studies have shown that the response to emotional stimuli is closely linked to conscious appraisal [18]. Therefore, in this sense, since the effect of emotions may be due to processing by the neurophysiological network employed to activate appraisal process, it is essential to also evaluate this aspect and not only the implicit appraisal response.

Considering these two approaches, it is therefore possible to interpret the results obtained from the abovementioned perspectives. Indeed, advertisements, on one hand, might automatically attract the attention of the audience as they are deemed more exciting than faces or body postures. However, on the other hand, people’s attention could be unconsciously captured by the contextual frame of the advertisement, other than faces or body postures. 

Therefore, beginning from this dual perspective, future research could better explore this issue by developing research protocols that manipulate the variables involved, such as, mimicry, posture, faces, etc., with respect to the advertising.

Some limitations should be considered in the present study. For instance, only the implicit dimension of the attitudes toward the brand was investigated (through IAT), and explicit judgements were not addressed. However, the literature has widely proven the existence of a significant gap between implicit and explicit attitudes [2]. Future research could include explicit measurements in order to better understand how explicit and implicit attitudes interplay in the consumer decision-making process. Additionally, emotional responses were analysed solely through neurophysiological markers. However, because of the multicomponent nature of emotion, future research could benefit from a multilevel approach encompassing an explicit assessment of the perceived emotional components and extending the recording of emotion-related neural processes to a wider neural network of areas which are known to contribute to the processing of emotions in decision-making beyond the PFC [5,88]. Furthermore, because emotions and emotion regulation are considered processes that unfold with specific temporal dynamics [89], additional insights could be gained by combing fNIRS with electroencephalography, which has been recently adopted to investigate neural patterns engaged in emotion processes with enhanced temporal resolution [90,91]. To comprehensively understand implicit processes and offer insight into the cognitive resources required for processing visual stimuli and their emotional impact on the audience, it might be desirable to conduct research using an eye-tracker. In fact, recording of patterns of fixations and saccades related to people’s eye movements in response to a visual stimulus can help researchers better understand how advertising affects users’ emotional excitement, visual attention, and cognitive workload [92,93]. In the meantime, these systems can be used to analyse programming techniques such as LINQ and algorithms or for the development of innovative software tools, providing, for example, tools for cognitive load or source code analysis or for describing readability evaluation algorithms. Along the same line of thinking, it might be interesting to implement research protocols that also include the use of immersive virtual reality to support vision screening while providing more emotional and interactive engagement [94]. Indeed, several studies showed how the use of these virtual immersive systems is a valuable tool for implementing a sense of self-confidence or self-efficacy [95,96]. Finally, future research should also better explore the valence effect related to COVID-19 and no-COVID-19 stimuli. Indeed, a possible interpretation of the present results could be supported by a more positive coping attitude induced by the sportswear stimuli, which induce a more “normal” post-crisis situation. 

Combining the current experimental multi-method protocol with future affective-focused assessments and interventions represents a possible potential future development of this study in both cognitive neuroscience and neuropsychology and in the clinical field. The first utilization of these research data certainly concerns the field of marketing, in which it is possible to implement the sale of a particular product and its recall by adopting high-impact advertising. In the meantime, since the results of this study revealed that stimuli with high emotional arousal are able to cognitively and emotionally engage the audience, it might be useful to use this type of communication in clinical and healthcare settings as well to increase, for example, patient engagement and disseminate correct prevention and treatment information. Moreover, another possible application could be inherent in the educational setting, where high-impact stimuli could be used to raise awareness and involvement on certain topics In conclusion, the present study was aimed at investigating implicit behavioural and neurovascular responses to emotionally arousing brand commercials that leveraged the COVID-19 pandemic. Our findings suggest that when initially presented, COVID-19-themed advertisements recruit the PFC to a greater extent than COVID-19-unrelated communications, in particular, recruiting the prefrontal cortices implied in emotion regulation and attentional resources allocation, presumably according to emotional salience. However, the results also highlight that the effectiveness of COVID-19 emotional stimuli may vanish when brand commercials (unrelated to COVID-19) are repeatedly shown prior to the COVID-19 advertisements, pointing out the role of presentation order. The behavioural results from the IAT point to the same conclusions since the IAT scores are consistent with the neurophysiological responses. Although further research is needed, the present findings may provide initial evidence in support of the role of prefrontal activity as a neural marker of implicit attitudes. Also, as emotional processing is not only subserved by the PFC, assessing the contribution of other cortical sites may prove constructive to achieve a deeper understanding.

## Data Availability

The datasets used and/or analysed during the current study are available from the corresponding author upon reasonable request.

## Supplementary Materials

The following supporting information can be downloaded at: https://www.mdpi.com/article/10.3390/s23094332/s1, Table S1, Summary of links to video advertisements shown during this study.
