# Supplementary material for: Implicit IAT Measures and Neurophysiological fNIRS Markers in Response to High-Engagement Advertising
Source: Sensors (Basel). 2023 Apr 27;23(9):4332. doi: 10.3390/s23094332 (PMC10181564; doi:10.3390/s23094332)
Supplement: Supplementary file 1 [file sensors-23-04332-s001.zip › sensors-2315822-supplementary.pdf]

Table S1. Summary of links to video advertisements shown during this study

	Title video	Link
**Video COVID-19-related**	*"Play for the World" (2020)*	https://www.youtube.com/watch?v=UFvCc7mxHMs (last accessed on 11 April 2023)
	"You Can't Stop LA"	https://www.youtube.com/watch?v=gWCdksLvtEw (last accessed on 11 April 2023)
	"You Can't Sop Us" (2020)	https://www.youtube.com/watch?v=pcXTnyCmQbg (last accessed on 11 April 2023)
**Video COVID-19-unrelated**	*"What's your motivation?"*	https://www.youtube.com/watch?v=6uhCxBk6cQE (last accessed on 11 April 2023)
	"You can't be stopped"	https://www.youtube.com/watch?v=2T3Wc9hsXB0 (last accessed on 11 April 2023)
	"Steps"	https://www.youtube.com/watch?v=lzUyaXTvmK4 (last accessed on 11 April 2023)
